# Application of organoid culture from HPV18‐positive small cell carcinoma of the uterine cervix for precision medicine

**DOI:** 10.1002/cam4.5588

**Published:** 2023-01-23

**Authors:** Misako Kusakabe, Ayumi Taguchi, Michihiro Tanikawa, Daisuke Hoshi, Saki Tsuchimochi, Xi Qian, Yusuke Toyohara, Akira Kawata, Ryota Wagatsuma, Kohei Yamaguchi, Yoko Yamamoto, Masako Ikemura, Kenbun Sone, Mayuyo Mori‐Uchino, Hiroko Matsunaga, Tetsushi Tsuruga, Takeshi Nagamatsu, Iwao Kukimoto, Osamu Wada‐Hiraike, Masahito Kawazu, Tetsuo Ushiku, Haruko Takeyama, Katsutoshi Oda, Kei Kawana, Yoshitaka Hippo, Yutaka Osuga

**Affiliations:** ^1^ Department of Obstetrics and Gynecology, Graduate School of Medicine The University of Tokyo Tokyo Japan; ^2^ Department of Molecular Carcinogenesis Chiba Cancer Center Research Institute Chiba Japan; ^3^ Division of Integrative Genomics, Graduate School of Medicine The University of Tokyo Tokyo Japan; ^4^ Department of Life Science and Medical Bioscience Waseda University Tokyo Japan; ^5^ CBBD‐OIL, AIST‐Waseda University Tokyo Japan; ^6^ Department of Surgical Oncology, Graduate School of Medicine The University of Tokyo Tokyo Japan; ^7^ Department of Pathology, Graduate School of Medicine The University of Tokyo Tokyo Japan; ^8^ Research Organization for Nano and Life Innovation Waseda University Tokyo Japan; ^9^ Pathogen Genomics Center National Institute of Infectious Diseases Tokyo Japan; ^10^ Division of Cellular Signaling National Cancer Center Research Institute Tokyo Japan; ^11^ Institute for Advanced Research of Biosystem Dynamics, Waseda Research Institute for Science and Engineering Waseda University Tokyo Japan; ^12^ Department of Obstetrics and Gynecology Nihon University School of Medicine Tokyo Japan

**Keywords:** cervical cancer, human papillomavirus, integration site, organoid, precision medicine

## Abstract

**Background:**

Small cell carcinoma of the uterine cervix (SCCC) is a rare and highly malignant human papillomavirus (HPV)‐associated cancer in which human genes related to the integration site can serve as a target for precision medicine. The aim of our study was to establish a workflow for precision medicine of HPV‐associated cancer using patient‐derived organoid.

**Methods:**

Organoid was established from the biopsy of a patient diagnosed with HPV18‐positive SCCC. Therapeutic targets were identified by whole exome sequencing (WES) and RNA‐seq analysis. Drug sensitivity testing was performed using organoids and organoid‐derived mouse xenograft model.

**Results:**

WES revealed that both the original tumor and organoid had 19 somatic variants in common, including the *KRAS* p.G12D pathogenic variant. Meanwhile, RNA‐seq revealed that HPV18 was integrated into chromosome 8 at 8q24.21 with increased expression of the proto‐oncogene *MYC*. Drug sensitivity testing revealed that a KRAS pathway inhibitor exerted strong anti‐cancer effects on the SCCC organoid compared to a MYC inhibitor, which were also confirmed in the xenograft model.

**Conclusion:**

In this study, we confirmed two strategies for identifying therapeutic targets of HPV‐derived SCCC, WES for identifying pathogenic variants and RNA sequencing for identifying HPV integration sites. Organoid culture is an effective tool for unveiling the oncogenic process of rare tumors and can be a breakthrough for the development of precision medicine for patients with HPV‐positive SCCC.

## Graphical abstract

We confirmed two strategies for identifying therapeutic targets of human papillomavirus (HPV)‐derived small cell carcinoma of the uterine cervix (SCCC), whole exome sequencing for identifying pathogenic variants and RNA sequencing for identifying HPV integration sites. Organoid culture is an effective tool for unveiling the oncogenic process of rare tumors and can be a breakthrough for the development of precision medicine for patients with HPV‐positive SCCC.

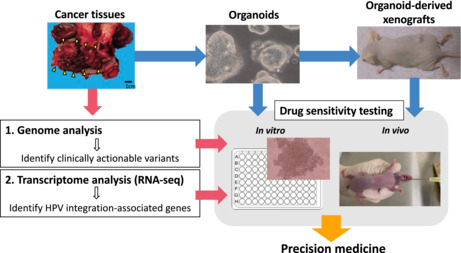

## 1 INTRODUCTION

Small cell carcinoma of the uterine cervix (SCCC) is a rare tumor, accounting for approximately 0.9% of all cervical cancers.^1^, ^2^ SCCC is a highly malignant tumor: it is usually detected at an advanced stage and the five‐year overall survival rates range from 14% to 67% for all stages, 30%–60% for early‐stage disease, and 0%–17% for an advanced‐stage disease which is worse than those for squamous cell carcinoma and adenocarcinoma.^1^ Although the aggressiveness of SCCC is similar to small cell carcinoma of other organs such as small cell lung cancer, its carcinogenesis is distinctive from that of other organs; human papillomavirus (HPV) infection is associated with its carcinogenesis.^3^ The genomic profile of SCCC is different from other HPV‐related cervical cancers; the most commonly detected oncogene mutations were PIK3CA (8/44), followed by KRAS mutations (6/44).^4^ Despite the introduction of HPV vaccination, cervical cancer (mainly caused by HPV infection) still remains the fourth most common cancer and the fourth leading cause of cancer death in women.^5^ In particular, SCCC is a rare and highly malignant tumor. Therefore, it is warranted to establish a workflow of precision medicine for SCCC based on its carcinogenesis.

With the continued advances in genomic technology, genome‐based precision medicine has become prevalent in several types of cancers; however, several challenges remain. One of the primary issues is that only a small number of patients can participate in genomically‐guided clinical trials. For instance, recent studies revealed that clinically actionable somatic mutations were identified in only approximately one‐third of patients,^6^ while only ~10% of patients who had undergone comprehensive genomic profiling were subsequently enrolled in genomically‐guided clinical trials.^7^, ^8^ Likewise, limitations exist when searching for clinically actionable therapeutic targets by DNA analysis alone. Therefore, it is necessary to search for therapeutic targets based on the characteristics of each type of carcinoma.

Human papillomavirus‐associated cancer involves multi‐step carcinogenesis. First, HPV‐derived E6 and E7 viral oncoproteins inactivate p53 and pRb tumor suppressor proteins, respectively, leading to resistance to apoptosis and promotion of cell proliferation.^9^ After persistent HPV infection, HPV DNA integration into the human genome can be a key step for the continuous expression of E6 and E7. In addition, HPV DNA integration triggers various genetic alterations, such as oncogene amplification, chromosomal rearrangement, and chromosomal instability.^10^, ^11^ Finally, the accumulation of DNA alterations in host cell genes is an important step in the progression of HPV‐infected cells to invasive cervical cancer. Hence, by taking into account HPV‐associated carcinogenesis, it may be possible to identify new therapeutic targets via analysis of HPV integration sites and subsequent genetic alterations.

Another challenge facing genome‐based precision medicine is that the therapeutic effects on individuals are unknown until the drug is administered. Therefore, it is important to establish preclinical models that can reliably reproduce intra‐ and inter‐tumor heterogeneity as well as the tumor microenvironment. In particular, for rare cancers, the development of patient‐derived models is important because of the difficulty of conducting large‐scale clinical trials. Meanwhile, organoid culture techniques emerged in the 2000s and have improved rapidly since the early 2010s.^12^ Patient‐derived organoids retain the morphology and expression patterns of their original organs. As such, organoids have been applied to various research fields, including tissue regeneration,^13^, ^14^ infectious disease,^15^ and malignant tumors.^16^, ^17^ By harnessing their ability to propagate normal tissues, we have established organoids from the cervical squamocolumnar junction, a specific target of HPV infection.^18^ Moreover, a previous report on cervical cancers has demonstrated that organoids retain the histological and transcriptional characteristics of the original cervical cancers.^19^ Their capacity to retain genetic and morphological similarities to their original tissues highlights the potential of organoids for precision medicine.

In order to establish a workflow of precision medicine for SCCC, we established an organoid line from SCCC and subjected it to drug sensitivity testing based on genetic information and HPV integration sites.

## 2 MATERIALS AND METHODS

### 2.1 Patients and clinical samples

The study was conducted in accordance with the Declaration of Helsinki. For the SCCC organoid, surgical specimens were obtained from a 26‐year‐old patient diagnosed with SCCC. To investigate MYC expression, HPV‐infected cervical cancer tissues were obtained from biopsy or surgical samples. Experienced pathologists confirmed the diagnosis according to pathological examination at the University of Tokyo Hospital. All the experimental procedures were approved by the Institutional Review Board at the University of Tokyo (approval number G0637‐12) and signed informed consent for the use of the tissues was obtained from each patient.

### 2.2 HPV genotyping

DNA was extracted from the cervical cancer tissues using the QIAamp DNA Mini Kit (QIAGEN), according to the manufacturer's instructions. HPV genotyping assays were performed using multiplex PCR, which is a rapid, high‐throughput genotyping procedure that allows the simultaneous detection of 16 genital HPV types,^20^ or by PCR with PGMY primers followed by reverse line blot hybridization that facilitates the detection of 31 HPV types.^21^

### 2.3 Primary organoid culture and passaging

**FIGURE 1:**
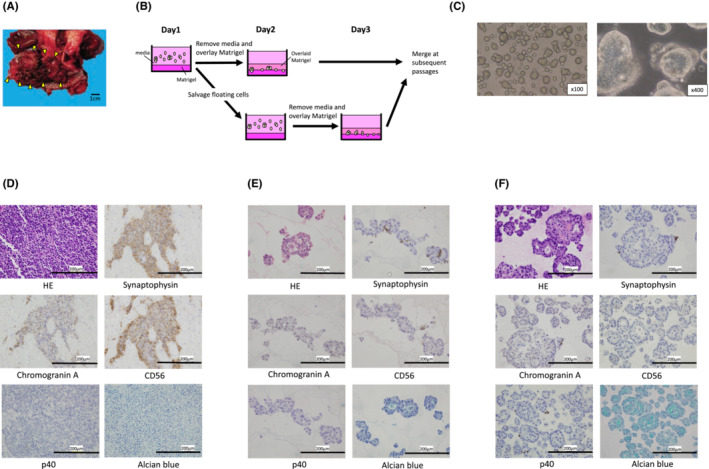
Established organoid maintains the histological architecture and neuroendocrine marker positivity of the original tumor. (A) Macroscopic view of a resected tumor (arrowheads) at the uterine cervix. (B) Simplified schematic representation of the modified Matrigel bilayer organoid culture protocol. (C) Established organoid of small cell carcinoma of the uterine cervix. (D–F) Histological examination of the original tumor (D) and organoid [(E): Passage 2, (F): Passage 4] by hematoxylin and eosin staining, immunohistochemical staining, and Alcian blue staining (×400).

The surgical biopsy sample from a patient with SCCC (Figure 1A) was cut into 2–3 mm pieces, washed with cold phosphate‐buffered saline (PBS) for several rounds and dissociated into small clusters or single cells using 2 U/ml dispase II (Wako), 1 mg/ml collagenase P (Roche Diagnostics) with 10 U DNase I (Sigma‐Aldrich) for 45 min at 37°C. They were further digested with Accumax (Innovative Cell Technologies) for 5 min at 37°C and washed with ice‐cold PBS. The organoid culture media was advanced Dulbecco's modified Eagle medium/F12 (#12634‐028; Thermo Fisher Scientific) supplemented with 50 ng/ml human epidermal growth factor, recombinant, Animal Free (#AF‐100‐15; Peprotech), 250 ng/ml Recombinant Mouse R‐spondin1 (#3474‐RS‐050; R&D), 100 ng/ml Noggin, Mouse (#250‐38; Peprotech), 10 μM Y27632 (073‐05391; Wako), 1 μM Jagged‐1 (#AS‐61298; AnaSpec), l‐glutamine solution (#073‐05391; Sigma‐Aldrich), penicillin–streptomycin‐amphotericin B suspension (Wako). The cells resuspended in 800 μl/well of the media were plated on 65 μl of solidified Matrigel (BD Biosciences) per well in a 12‐well plate and incubated overnight at 37°C. The following day, cells attached to the Matrigel were covered with extra 70 μl of Matrigel and overlaid with 800 μl/well of the media. To increase the tumor cell yield, we further digested the floating tissue fragments and cell aggregates using Accumax, followed by primary organoid culture performed according to the modified Matrigel bilayer organoid culture (MBOC) protocol (Figure 1B).^22^ The media was changed every 2–3 days, and passage was conducted at 70%–80% confluency. The cells were diluted at 1:4 to 1:6. In each passage, organoids, Matrigel, and the media were directly collected altogether with a cell scraper, washed with PBS, and dissociated into single cells, by Accumax treatment for 5 min at 37°C and pipetting.

### 2.4 Pathological analyses of the original tumor and organoid

Organoid pellets were prepared by dissolving Matrigel with cell recovery solution (Corning) at 4°C for approximately 30 min. To create an organoid cell block, the prepared organoid pellet was solidified using iPGell (Genostaff), according to the manufacturer's instructions. Immunohistochemistry of each marker was performed using organoid cell blocks and organoid‐derived mouse xenograft. The detailed method is described in the Supplementary Methods.

### 2.5 DNA extraction, library preparation, and whole exome sequencing

DNA was extracted from the cultured organoids (passage 5) and resected fresh frozen tumor using the QIAamp DNA Mini Kit (QIAGEN), according to the manufacturer's instructions. DNA purity and concentration were determined using a NanoDrop 2000 spectrophotometer (Thermo Fisher Scientific, Inc.). For the library preparation, the SureSelect Human All Exon V6 Kit (Agilent Technologies) was used, and samples were sequenced using a NovaSeq 6000 sequencer at 2 × 150 bp (Illumina). Sequence alignment and mutation calling were performed as previously described.^23^ Read mapping and variant calling are described in the Supplementary Methods.

### 2.6 Copy number variation

The copy number variation (CNV) profiles of the original tumor and organoids were created using WES. The pileup file was generated from the processed BAM file using FACETS (ver. 0.6.0),^24^ a specific copy number analysis tool for high‐throughput DNA sequencing.

### 2.7 RNA sequencing and RNA‐seq data analysis

RNA sequencing (RNA‐seq) was used to compare the HPV18‐derived gene expression patterns in the original tumor and the established organoids. Total RNA extraction, preparation of the cDNA library, RNA‐seq, and data analysis were performed as described previously.^25^ Human‐virus fusion mRNA was detected by Viral Integration and Fusion Identification (ViFi).^26^, ^27^ These procedures are described in the Supplementary Methods.

### 2.8 Validation of the integration site by PCR

Total RNA was extracted from organoids and fresh frozen tissues using the miRNeasy mini kit (QIAGEN), according to the manufacturer's instructions. After estimating the integration site based on the data obtained with the ViFi tool (Table S1), primers for HPV18 and human chromosome 8 were designed to validate the integration site using Primer3 v.0.4.0,^28^ Primer‐BLAST,^29^ and Multiple Primer Analyzer^30^: HPV18: 5′‐CACGAGCAATTAAGCGACTCAG‐3′; human: 5′‐GCACATGTGGACCAAAAGACA‐3′. cDNA was synthesized using 4 μg of total RNA and a PrimeScript™ IV 1st strand cDNA Synthesis Mix with random hexamer (Takara Bio, Inc.). For strand‐specific PCR, strand‐specific primers were used instead of a random hexamer. After amplification for 30 cycles in the Gene Atlas G02 Gradient Thermal Cycler System (ASTEC, Fukuoka, Japan), the PCR products were resolved using agarose gel electrophoresis, cloned, and then sequenced.

### 2.9 Real‐time quantitative PCR of MYC

Total RNA was extracted from organoids, fresh frozen tissues, and cell lines using an RNeasy mini kit (QIAGEN), according to the manufacturer's instructions. The ratio of absorbance at 260 and 280 nm was evaluated using a NanoDrop 2000 spectrophotometer (Thermo Fisher Scientific, Inc.), and a ratio of more than 2.0 was used in the study. cDNA was synthesized using 4 μg of total RNA and a PrimeScript IV first strand cDNA Synthesis Mix with random hexamers (Takara Bio, Inc.). cDNA was amplified with KOD SYBR^®^ qPCR Mix (TOYOBO Co., Ltd.) and the following gene‐specific primer pairs: 5′‐CAGCTGCTTAGACGCTGGATTT‐3′ and 5′‐ACCGAGTCGTAGTCGAGGTCAT‐3′.^31^ All targets were amplified (30 cycles) using a Quant Studio1 real‐time PCR system (Thermo Fisher Scientific, Inc.). The expression levels were calculated using the 2^−ΔΔCT^ method with *ACTB* (β‐actin) serving as the endogenous reference gene.

### 2.10 Drug sensitivity testing in vitro

Organoids were collected and dissociated into single cells by Accumax digestion and counted using a cell counter (Olympus). The detailed method is described in the Supplementary Methods.

### 2.11 Analysis of cell cycle by flow cytometry

The method is described in the Supplementary Methods.

### 2.12 Establishment of organoid‐derived mouse xenograft models and drug sensitivity testing in vivo

Animal studies were conducted with the approval of the Animal Care and Use Committee of the University of Tokyo in compliance with the institutional guidelines, following the ARRIVE guidelines 2.0. Details are described in the Supplementary Methods.

## 3 RESULTS

### 3.1 Establishment of the SCCC organoid with neuroendocrine features

Cervical cancer samples were obtained from surgical biopsies (Figure 1A). Cells were plated onto Matrigel using a modified MBOC method (Figure 1B). The SCCC organoid exhibited small round morphologies in the early passages (Figure 1C); however, after several passages, cystic parts emerged from the small round structures. The original tumor was histologically pure SCCC, positive for neuroendocrine markers such as synaptophysin, chromogranin A, and CD56, and negative for Alcian blue staining (Figure 1D). Histologically, established organoids showed glandular structures accompanied by intracellular mucin production, suggesting glandular differentiation had occurred. Immunohistochemically, at the early passages, cells expressing neuroendocrine markers, including synaptophysin, chromogranin A, and CD56, were observed to be sparsely present; however, these cells decreased during repeated passages (Figure 1E,F). In contrast, Alcian blue staining became diffusely positive in the organoids after several passages, supporting the glandular differentiation of the organoids. In addition, cells positive for a squamous cell carcinoma marker, p40, appeared sparsely after repeated passages.

### 3.2 Identification of clinically actionable targets by genomic analysis

**FIGURE 2:**
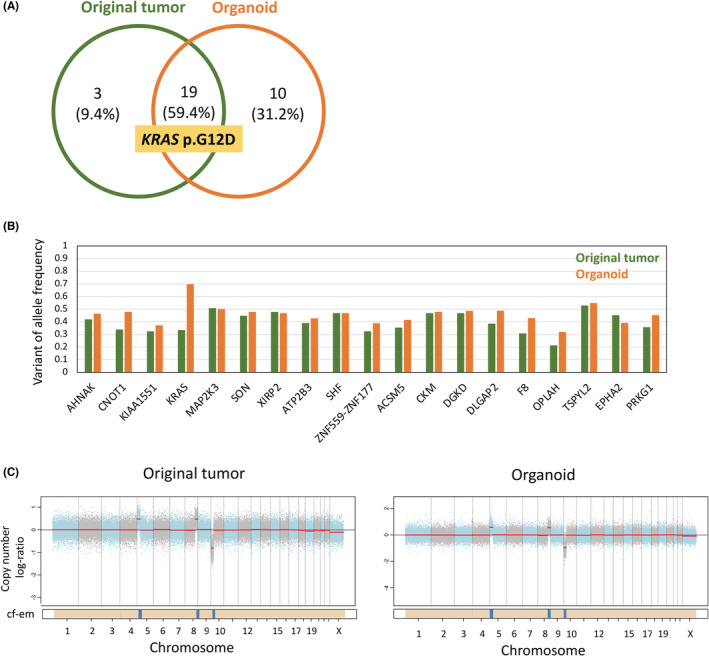
Organoid‐replicated genomic landscape of the primary tumor. (A) Somatic mutation profiles by whole exome sequencing in the original tumor and organoid. (B) Comparison of VAF of genetic alterations detected in the original tumor and organoid. (C) Copy number variation profiles of the original tumor and organoid. The panel displays the total copy number log ratio with chromosomes alternating in blue and gray. The median log ratio of each segment is shown in red. The purple line indicates the log ratio of the diploid state. The bottom bar shows the associated cellular fraction (cf). Dark blue indicates high cf. Beige indicates a normal segment. Cf‐em: the cellular fraction of the segment.

Whole exome sequencing was used to identify therapeutic targets and investigate clonal changes in organoid cultures. WES revealed 22 and 29 somatic single nucleotide variants or intermediate long insertions/deletions in the original tumor and the established SCCC organoid, respectively (Table S2). Among them, 19 variants, including the *KRAS* p.G12D pathogenic variant, co‐existed in the original tumor and organoid (Figure 2A). Although the variant allele frequencies (VAFs) of common variants were similar between the organoid and primary tumors, the VAF of the *KRAS* variant was doubled in the organoid (Figure 2B). Subsequently, we conducted CNV analysis and observed that both the original tumor and the SCCC organoid had copy number gains in chromosome 5 (5p12‐15.33) and 8 (8q24.1‐24.23), as well as copy number losses in chromosome 10 (10p11.23‐15.3) (Figure 2C; Table S3).

### 3.3 Identification of HPV18 integration sites into the human genome

**FIGURE 3:**
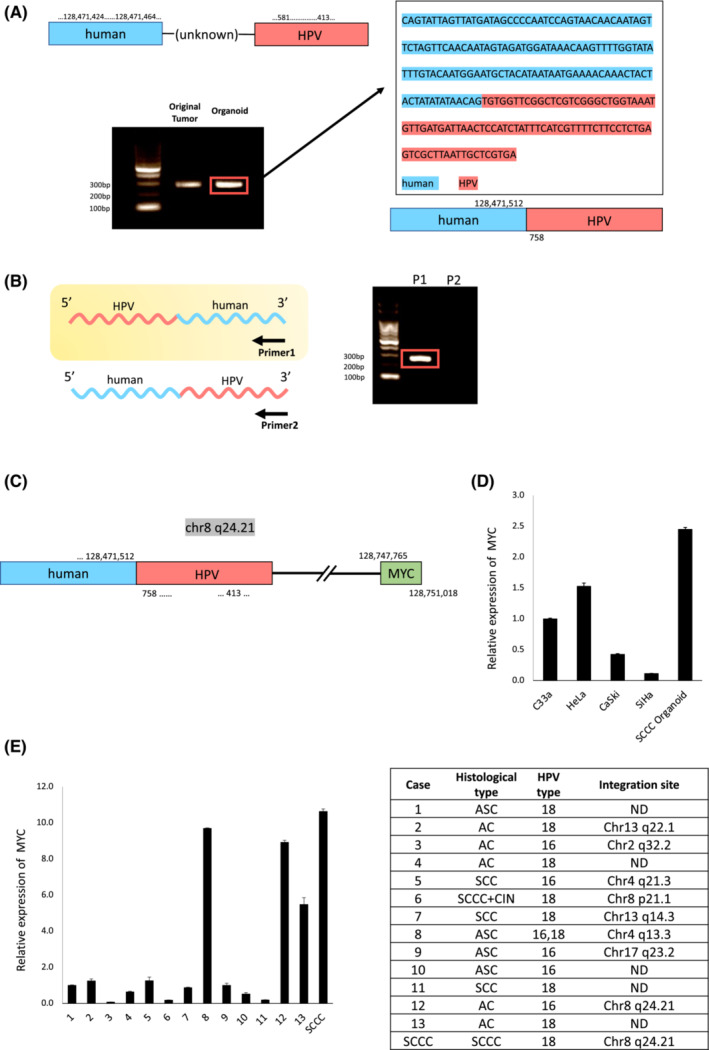
Validation of the integration site. (A) The integration site was estimated by the ViFi tool, and the viral‐human fusion sequence was validated by PCR using designed primers. The PCR products were analyzed by 2% agarose gel electrophoresis, cloned, and then sequenced. (B) Validation of the orientation of viral‐human fusion by strand‐specific PCR. cDNA synthesis was performed using strand‐specific primers (Primer 1: primer of human side, Primer 2: primer of HPV18 side) and amplified by PCR. (C) Identified structure of the integration site. (D) Comparison of *MYC* expression between the SCCC organoid and four cervical cancer cell lines, i.e., C33a (HPV‐negative), HeLa (HPV18‐positive), CaSki (HPV16‐positive), SiHa (HPV16‐positive), evaluated by RT‐qPCR analysis using the delta–delta Ct method. (E) Comparison of *MYC* expression between the original SCCC tissue and 13 HPV‐associated cervical cancer some cases of which HPV integration sites were identified. The table on the right shows the integration site for each case estimated by the ViFi tool. AC, adenocarcinoma; ASC, adenosquamous carcinoma; CIN, cervical intraepithelial neoplasia; HPV, human papillomavirus; ND, not determined; SCC, squamous cell carcinoma; SCCC, small cell carcinoma of the uterine cervix; ViFi, Viral Integration and Fusion Identification

To assess the HPV18‐derived transcriptome and viral‐human chimeric RNAs, RNA‐seq analysis was performed. Sequences mapped to the HPV18 reference were visualized using Integrative Genomics Viewer (IGV) version 2.9.4.^32^ The expression of HPV18‐derived transcripts was similar between the original tumor and the SCCC organoid (Figure S1). However, differences were observed in the expression patterns of HPV18 E7, i.e., an interruption of the transcription product in the middle of E7 (nt759) was more prominent in the SCCC organoid than the original tumor (Figure S1). Subsequently, we investigated viral‐human chimeric RNAs using ViFi. Two types of chimeric RNAs were identified in the original tumor, one of which was identified in the SCCC organoid (Table S1). We validated the detected chimeric RNA by PCR (Figure 3A–C). Sequence data of the amplified PCR products showed that HPV18 was integrated into chromosome 8:128,471,512 (8q24.21) (Figure 3A). Strand‐specific PCR revealed that the human sequence was located at the 5′ end of the chimeric RNA and the HPV sequence at the 3′ end. Additionally, the chimeric RNA was located on the reverse strand (Figure 3B).

### 3.4 Expression of MYC is upregulated in cervical cancers with HPV integration into chromosome 8q24.21

In this case, the proto‐oncogene *MYC* was located approximately 280 kb downstream of the identified integration site (8q24.21). To assess the association between HPV integration sites and MYC expression, MYC mRNA expression was compared between the SCCC organoid and four cervical cancer cell lines, namely C33a (HPV‐negative), HeLa (HPV18‐positive), CaSki (HPV16‐positive), and SiHa (HPV16‐positive) (Table S4). Compared to the CaSki and SiHa cells, HeLa cells containing the HPV18 integration site upstream of *MY*C (8q24.21) exhibited MYC upregulation.^33^ The MYC levels in the SCCC organoid were even more elevated (Figure 3D). Furthermore, MYC expression was compared between the original SCCC tissue and 13 HPV‐associated cervical cancers; their HPV integration sites were analyzed by RNA‐seq (Table S5). Among them, HPV genome integration into the 8q24.21 site was detected in the SCCC case and Case 12, with MYC overexpression observed in both of them (Figure 3E).

### 3.5 A KRAS pathway inhibitor exerts strong anti‐cancer effects on the SCCC organoid in vitro

**FIGURE 4:**
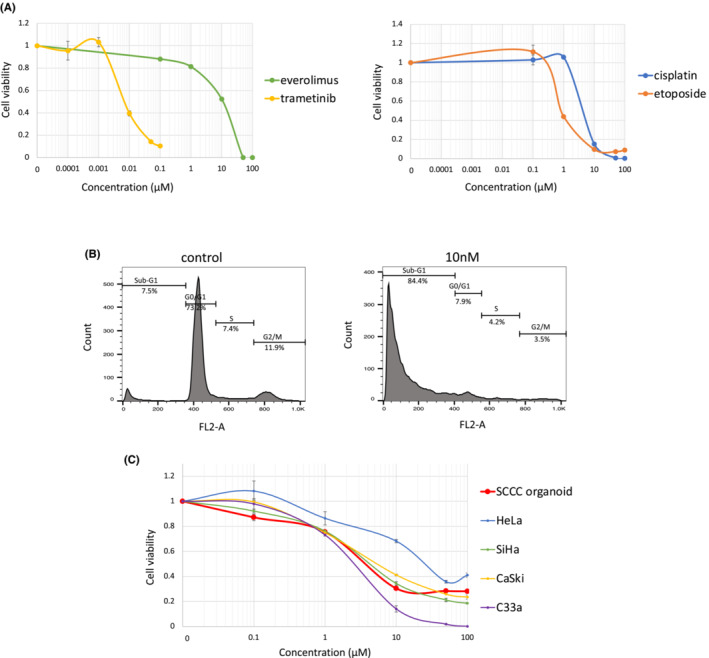
Drug sensitivity testing of the SCCC organoid to MEK inhibitor and MYC inhibitor. (A) Dose–response curves of the SCCC organoid treated with trametinib and everolimus (left graph), cisplatin, and etoposide (right side). Mean ± SD of the technical triplicates is shown for each drug. (B) SCCC organoids were treated with trametinib at 10 nM for 72 h (control = 0.4% dimethyl sulfoxide), followed by staining with propidium iodide for cell cycle distribution, and then analyzed using flow cytometry. Three independent experiments were performed. (C) Dose–response curves of the SCCC organoid and cervical cancer cell lines treated with MYCi975. Mean ± SD of the technical triplicates is shown for each drug. SCCC, small cell carcinoma of the uterine cervix; SD, standard deviation

Considering that the existence of *KRAS* pathogenic variants results in activation of the intracellular RAS–RAF–MEK–ERK pathway, mitogen‐activated extracellular signal‐regulated kinase (MEK) inhibitors are expected to be key drugs for the treatment of *KRAS*‐mutant cancers. Therefore, in addition to the commonly used chemotherapy, cisplatin and etoposide, we investigated the effect of trametinib, a MEK inhibitor, on the viability of SCCC organoids using drug sensitivity testing. Everolimus, an mTOR inhibitor, was used as a negative control. The drug sensitivity testing showed that the established SCCC organoid was partially sensitive to cisplatin and etoposide with IC_50_ values of 4.1 and 0.82 μM, respectively. In contrast, trametinib markedly reduced the number of viable SCCC cells with an IC_50_ of 6.8 nM (Figure 4A). Cell cycle analysis revealed that treatment with trametinib induced a significant increase in cells in the sub‐G1 phase, while decreasing cells in the G0/G1, S, and G2/M phases (Figure 4B), suggesting that trametinib induced apoptosis.

### 3.6 A MYC inhibitor suppresses cell viability of SCCC organoids, however, its effect does not correlate with MYC expression

With an observed increase in MYC expression, a MYC inhibitor was proposed as another molecular target drug against SCCC. Subsequently, we investigated the effect of MYCi975, a MYC inhibitor, on the viability of SCCC organoids by drug sensitivity testing. MYCi975 suppressed the viability of SCCC organoid cells with an IC_50_ of 3.7 μM, which is comparable to the cell sensitivity of MYC‐dependent cell lines reported previously (Figure 4C).^34^ Subsequently, to assess the association between MYC expression levels and cell sensitivity to the MYC inhibitor, the cell sensitivity of SCCC organoids was compared to other cervical cancer cell lines, HeLa, CaSki, SiHa, and C33a. However, sensitivity to MYCi975 did not correlate with MYC expression level (Figures 3D and 4C).

### 3.7 A KRAS pathway inhibitor suppresses tumor growth in vivo in the organoid‐derived mouse xenograft model

**FIGURE 5:**
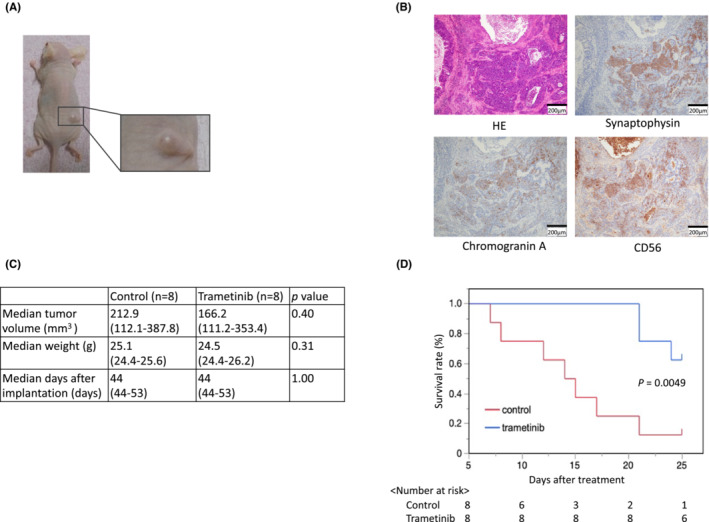
Drug sensitivity testing of the small cell carcinoma of the uterine cervix organoid to MEK inhibitor. (A) Established organoid‐derived mouse xenograft mouse model. (B) Histological examination of the organoid‐derived mouse xenograft. (C) The median tumor volume and weight at the beginning of the treatment and days after implantation in each group were not significantly different. (D) Kaplan–Meier curve between a control group and a trametinib‐treated group.

From the in vitro drug sensitivity testing, trametinib was selected as a therapeutic candidate of the SCCC case. To assess the anti‐cancer effect of trametinib in vivo, we conducted in vivo drug sensitivity testing by establishing a organoid‐derived mouse xenograft model (Figure 5A). First, we conducted histological evaluation of organoid‐derived mouse xenograft and confirmed the increased proportion of small cell carcinoma components (Figure 5B). Upon initiation of the treatment, no significant differences were observed in median tumor volume, body weight, and days after implantation between the two groups (Figure 5C). In the control group, 7/8 mice achieved volumes of 500 mm^3^, with a median survival time of 14.5 days. In contrast, 3/8 mice in the trametinib group achieved volumes of 500 mm^3^. Thus, tumor growth was significantly suppressed in the trametinib group compared to the control group (*p* = 0.0049; Figure 5D).

## 4 DISCUSSION

In this study, we established an organoid line from SCCC, a rare and highly malignant cancer. Genomic analysis of the established organoid identified a clinically actionable somatic variant (*KRAS* p.G12D). In addition, investigation of viral‐human chimeric RNA revealed that HPV18 was integrated into chromosome 8 (8q24.21) accompanied by copy number gain of this cytoband and increased expression of the proto‐oncogene *MYC*. In vitro drug sensitivity testing using an established SCCC organoid revealed that among the two therapeutic targets of precision medicine identified, the KRAS pathway inhibitor exerted adequate anti‐cancer effects compared to the MYC inhibitor. In addition, the anti‐cancer effect of trametinib was also confirmed by the organoid‐derived mouse xenograft model.

*KRAS* p.G12D is one of the most frequently detected *KRAS‐*activating mutations, which leads to continuous activation of downstream pathways.^35^ *KRAS* mutations are detected in 0%–1.3% of cervical squamous cell carcinoma and in 8.3%–17.5% of cervical adenocarcinoma.^11^, ^36^, ^37^ Although information on *KRAS* mutations in SCCC is limited due to its rarity, a study of 44 SCCC patients reported that 14% had *KRAS* mutations in their tumors.^4^ As the development of specific and direct KRAS inhibitors has been difficult, due to the absence of an ideal binding pocket for a small, structurally dynamic protein with a strong affinity for GTP,^38^ inhibitors of MEK, an effector located downstream of *KRAS*, have attracted attention as agents to inhibit *KRAS*‐dependent tumorigenesis. In the present case, the MEK inhibitor markedly suppressed the proliferation of SCCC organoids, suggesting that they induced apoptosis, thus, confirming the possibility of using a MEK inhibitor as a therapeutic candidate for genome‐based precision medicine.

Overexpression of MYC is frequently found in HPV‐infected cervical cancer.^39^ Being located on chromosome band 8q24.21, its overexpression is sometimes related to HPV integration into the flanking region.^10^, ^40^, ^41^ In HPV18‐positive HeLa cells, the HPV18 genome is integrated into 8q24.21, accompanied by elevated expression of the MYC protein.^40^ Likewise, *MYC* is a noteworthy target, particularly in HPV‐related cervical cancers. Although upregulation of MYC is prevalent in HPV‐associated cervical cancer, MYC‐targeted therapy remains a challenge for a couple of reasons. First, MYC protein lacks a readily identifiable accessible deep pocket into which potential low molecular weight drugs can bind with high affinity. Second, MYC has no enzymatic activity. Therefore, it cannot be targeted by low molecular weight catalytic inhibitors.^42^ In this study, a MYC inhibitor exerted an effect on SCCC organoids; however, this effect was independent of MYC expression levels, making the usage of MYC expression as a biomarker for therapeutic drug selection difficult.

HPV integration is found in 53.8% of cervical intraepithelial neoplasia and 81.7% of cervical cancers.^10^ In HPV‐associated cancer, HPV DNA integration triggers various genetic alterations, such as oncogene amplification, chromosomal rearrangements, and chromosomal instability. In addition, HPV integration generates a super‐enhancer‐like element to drive high levels of oncogene expression.^43^ Therefore, the identification of human genes related to the integration site may represent another target for precision medicine. Other than *MYC*, *HMGA2* is reportedly upregulated in uterine cervical cancer when integrated into flanking regions.^10^ This strategy can apply not only to cervical cancer but also to other HPV‐related tumors. For example, in the HPV‐positive head and neck cancer, *NR4A2*, an orphan nuclear receptor, is upregulated when HPV is integrated upstream of *NR4A2*.^44^ Hence, in HPV‐related cancers, the identification of integration sites, and associated genetic alterations may increase the opportunities for effective precision medicine.

As a strategy to assess responses to specific drugs, organoid technology has shown tremendous potential for applications in precision medicine. Organoids can be rapidly generated with small tumor samples and recapitulate the characteristics of their original tissues. In the current study, although established organoids exhibited glandular differentiation over several passages, the established organoid had similar genomic profiles and the same HPV integration site as the original tumor. In addition, an in vivo evaluation of the organoid‐derived xenograft showed that the expression of neuroendocrine markers recovered, indicating the restoration of small cell carcinoma characteristics. Thus, it is important to combine in vitro screening and in vivo validation; the in vitro drug sensitivity testing was used to screen for drugs that would have a therapeutic effect and the in vivo drug sensitivity testing, in which the phenotype also returns to the original tumor, was used as a validation model.

This study has several limitations. First, although organoid culture generally reflects biological conditions efficiently, repeated propagation leads to alterations in cellular differentiation. In this case, the resected tumor was originally a pure small cell carcinoma; however, glandular differentiation was observed during the process of organoid culture. Therefore, drug sensitivity does not necessarily affect pure small cell carcinoma. However, considering that a pivotal driver mutation was maintained after several passages, this organoid model may be applicable for cancer precision medicine. In addition, we confirmed that organoid‐derived mouse xenograft recovered small cell carcinoma characteristics, therefore, organoids can be useful for drug screening while maintaining similar genetic characteristics to the original tumor, and then in vivo drug sensitivity assay can be useful to validate the therapeutic effects on tumors with same histological features. Second, there are several hot spots of HPV integration sites detected in HPV‐related cancers,^45^ and therapeutic targets can differ depending on the integration site.^40^, ^46^ In the current case, since the HPV integration site was close to the integration site of HeLa cells, we were able to identify *MYC* as a therapeutic target. To exploit HPV integration site‐based precision medicine, it may be important to identify candidates for therapeutic targets at each hot spot.

**FIGURE 6:**
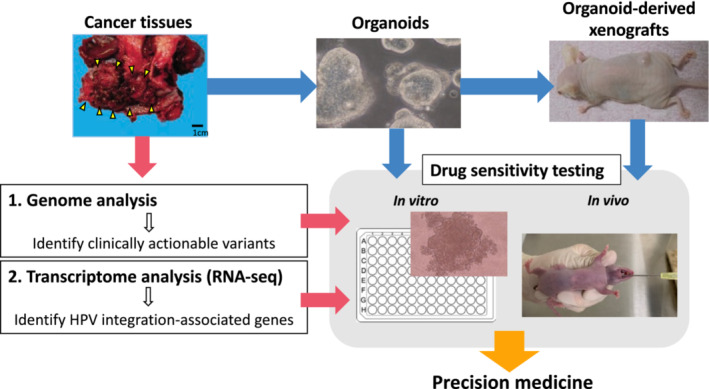
Workflow of our research.

Despite the several limitations, herein, we proposed a novel workflow for precision medicine for HPV‐derived cancers (Figure 6). Therapeutic targets can be identified by two strategies, genome analysis (for identifying pathogenic variants) and transcriptome analysis (for identifying HPV integration‐related oncogene activation). Therapeutic targets focused on integration sites can be more useful when multigene panel testing fails to find a target. The organoid culture models were used to assess the therapeutic effects of the identified targets. By focusing on HPV integration sites as well as genomic alterations, a possible opportunity arises to provide precision medicine to a greater number of patients with cervical cancer.

## AUTHOR CONTRIBUTIONS

**Misako Kusakabe:** Data curation (lead); investigation (lead); methodology (lead); visualization (lead); writing – original draft (lead); writing – review and editing (equal). **Ayumi Taguchi:** Conceptualization (lead); data curation (equal); funding acquisition (lead); investigation (supporting); methodology (equal); project administration (equal); writing – original draft (supporting); writing – review and editing (equal). **Michihiro Tanikawa:** Project administration (equal); writing – review and editing (supporting). **Daisuke Hoshi:** Methodology (supporting); resources (lead); writing – review and editing (supporting). **Saki Tsuchimochi:** Investigation (supporting); writing – review and editing (supporting). **Xi Qian:** Investigation (supporting); writing – review and editing (supporting). **Yusuke Toyohara:** Investigation (supporting); writing – review and editing (supporting). **Akira Kawata:** Methodology (supporting); writing – review and editing (supporting). **Ryota Wagatsuma:** Data curation (supporting); formal analysis (equal); investigation (supporting); software (equal); writing – review and editing (supporting). **Kohei Yamaguchi:** Writing – review and editing (supporting). **Yoko Yamamoto:** Methodology (supporting); resources (equal); writing – review and editing (supporting). **Masako Ikemura:** Writing – review and editing (supporting). **Kenbun Sone:** Writing – review and editing (supporting). **Mayuyo Mori‐Uchino:** Writing – review and editing (supporting). **Hiroko Matsunaga:** Data curation (supporting); formal analysis (equal); investigation (supporting); software (equal); writing – review and editing (supporting). **Tetsushi Tsuruga:** Writing – review and editing (supporting). **Takeshi Nagamatsu:** Writing – review and editing (supporting). **Iwao Kukimoto:** Investigation (supporting); writing – review and editing (supporting). **Osamu Wada‐Hiraike:** Supervision (supporting); writing – review and editing (supporting). **Masahito Kawazu:** Data curation (equal); formal analysis (equal); investigation (supporting); software (equal); writing – review and editing (supporting). **Tetsuo Ushiku:** Supervision (equal); writing – review and editing (supporting). **Haruko Takeyama:** Supervision (equal); writing – review and editing (supporting). **Katsutoshi Oda:** Supervision (equal); writing – review and editing (supporting). **Kei Kawana:** Supervision (equal); writing – review and editing (supporting). **Yoshitaka Hippo:** Methodology (supporting); supervision (equal); writing – review and editing (supporting). **Yutaka Osuga:** Supervision (equal); writing – review and editing (supporting).

## FUNDING INFORMATION

This study was supported by a grant to A.T. by AMED (Grant Number: 22wm0325014h0003) and JSPS KAKENHI (Grant Number: 20K18157). This research was partially supported by Platform Project for Supporting Drug Discovery and Life Science Research (Basis for Supporting Innovative Drug Discovery and Life Science Research; BINDS) from AMED (Grant Number: JP21am0101104). The funders did not have any role in study design, analyses, interpretation of the data, or preparation of the manuscript.

## CONFLICT OF INTEREST

The authors have no conflict of interest to declare.

## ETHICS STATEMENT

Approval of the research protocol by an Institutional Reviewer Board. All the experimental procedures were approved by the Institutional Review Board at the University of Tokyo. *Informed consent*: Signed informed consent for the use of the tissues was obtained from each patient. *Registration no. of the study*: G0637‐12. *Animal studies*: Animal studies were conducted with the approval of the Animal Care and Use Committee of the University of Tokyo in compliance with the institutional guidelines.

## Supporting information


Appendix S1.

Figure S1.

Table S1.

Table S2.

Table S3.

Table S4.

Table S5.

## Data Availability

Sequence data generated in this study has been deposited at the Japanese Genotype‐phenotype Archive (JGA, https://www.ddbj.nig.ac.jp/jga), which is hosted by the Bioinformation and DDBJ Center, under accession number JGAS000586.
