# Supplementary material for: Application of organoid culture from HPV18‐positive small cell carcinoma of the uterine cervix for precision medicine
Source: Cancer Med. 2023 Jan 23;12(7):8476–89. doi: 10.1002/cam4.5588 (PMC10134306; doi:10.1002/cam4.5588)
Supplement: Supplementary file 1 — Appendix S1. [file CAM4-12-8476-s002.docx]

**Supplementary Methods**

**Application of organoid culture from HPV18-positive small cell carcinoma of the uterine cervix for precision medicine**

Misako Kusakabe, Ayumi Taguchi, Michihiro Tanikawa, Daisuke Hoshi,

Saki Tsuchimochi, Xi Qian, Yusuke Toyohara, Akira Kawata, Ryota Wagatsuma, Kohei Yamaguchi, Yoko Yamamoto, Masako Ikemura, Kenbun Sone, Mayuyo Mori-Uchino, Hiroko Matsunaga, Tetsushi Tsuruga, Takeshi Nagamatsu, Iwao Kukimoto, Osamu Wada-Hiraike, Masahito Kawazu, Tetsuo Ushiku, Haruko Takeyama, Katsutoshi Oda, Kei Kawana,

Yoshitaka Hippo, Yutaka Osuga

**Pathological analyses of original tumor, patient-derived organoid and organoid-derived xenografts**

The original tumor, iPGell-embedded organoids and organoid-derived xenografts were fixed with 10% neutral buffered formalin. They were then dehydrated, embedded in paraffin, and sectioned at 4 μm for the original tumor and 5 μm for the organoid. The sections were deparaffinized and stained with hematoxylin and eosin. Primary antibodies against the following proteins were used for immunohistochemical studies of the original tumor and iPGell-embedded organoids: synaptophysin (MRQ-40, Sigma-Aldrich, St. Louis, MO, USA), chromogranin A (M0869, DAKO, Santa Clara, CA, USA), CD56 (418191, Nichirei Bioscience, Tokyo, Japan), and p40 (418171, Nichirei Bioscience, Tokyo, Japan). And for immunohistochemical studies of organoid-derived xenografts: synaptophysin (17785-1-AP, ProteinTech Group, Chicago, IL, USA), chromogranin A (M0430, DAKO, Santa Clara, CA, USA), and CD56 (NCL-CD56-1B6, Leica Biosystems, Nussloch, Germany). To evaluate mucin histochemistry, the original tumor and the organoid were stained with Alcian blue (FUJIFILM Wako Pure Chemical Corporation, Osaka, Japan).

**Read mapping and variant calling**

The sequence reads were mapped to the human reference genome GRCh37/hg19 using Burrows–Wheeler Aligner (v0.7.10). Possible PCR duplicates, read pairs with a mapping quality of <30, and mismatches of >5% were excluded. Somatic variants were called using Fisher’s exact test-based methods in accordance with the following parameters: (1) base quality of  ≥15, (2) sequence depth of  ≥10, (3) variant depth of  ≥4, (4) variant frequency in tumors of  ≥10%, (5) variant frequency in normal samples of <2%, and (6) Fisher *P*-value  <0.05.

**Total RNA extraction, preparation of the cDNA library, RNA sequencing (RNA-seq)**

Total RNA was extracted from fresh-frozen tumor samples and organoid pellets using the RNeasy mini kit (QIAGEN, Hilden, Germany) according to the manufacturer’s instructions and stored at -80 °C. The RNA integrity number equivalent (RINe) was measured using a Tapestation 4200 (Agilent, Tokyo, Japan) and RNA concentration was measured using the Qubit RNA HS Assay Kit (Thermo Fisher Scientific Inc., Waltham, MA, USA). For cDNA library preparation, 500 pg of total RNA was used. First, mRNA purification was performed using ProK (QIAGEN, Hilden, Germany) treatment and oligo(dT) magnetic beads (Thermo Fisher Scientific Inc., Waltham, MA, USA), followed by cDNA library preparation according to the SMART-seq2 protocol.^1^ Amplified cDNA products were purified with 0.8× volume of AMPure XP beads (Beckman Coulter, Brea, CA, USA). Purified cDNAs were used for sequencing library preparation by the Nextera XT DNA library prep kit (Illumina Inc., San Diego, CA, USA). The libraries were sequenced with 75 bp paired-end read on an Illumina Miseq (Illumina Inc., San Diego, CA, USA).

**RNA-seq data analysis**

The adapter sequences were trimmed off from the raw reads data using flexbar (ver. 3.5.0). Human-virus fusion mRNA was detected by ViFi: Viral Integration and Fusion Identification. The gene expression levels of HPV18 were calculated using VIRTUS pipeline (ver. 1.2), and the sequences mapped to the HPV18 reference (downloaded from PaVE; available at [https://pave.niaid.nih.gov/](about:blank)) were visualized using the Integrative Genomic Viewer (IGV) program (available at https://software.broadinstitute.org/software/igv/).

**Drug sensitivity testing *in vitro***

*Determining the efficacy of trametinib (a mitogen-activated extracellular signal-regulated kinase inhibitor)*

For drug sensitivity testing, 5 × 10^3^ single cells/well were plated into PrimeSurface 96U (Sumitomo Bakelite, Tokyo, Japan) in triplicate. At 48 hours after plating, cisplatin (Nichi-Iko Pharmaceutical Co., Ltd., Toyama, Japan), etoposide (E1383-25MG, Sigma-Aldrich, St. Louis, MO, USA), trametinib (GSK1120212, Selleckchem, Houston, TX, USA) and everolimus (LC Laboratories, Woburn, MA, USA) were added in five serially diluted doses from 0.1 to 100 μM, from 0.1 to 100 μM, from 0.1 to 100 nM, and from 0.1 to 100 μM, respectively. The cell viability was analyzed using CellTiter‐Glo3D Cell Viability Assays (Promega, Fitchburg, WI, USA) in triplicate following 96 h of drug incubation. The half-maximal inhibitory concentration (IC_50_) of trametinib was compared with that of cisplatin and etoposide, which are commonly used in clinical settings for SCCC. Everolimus, an mTOR inhibitor, was used as a negative control.

*Determining the efficacy of MYCi975 (a MYC inhibitor)*

The organoids were seeded as described above. The HeLa, SiHa, CaSki, and C33a cell lines, purchased from American Type Culture Collection (ATCC, Manassas, VA, USA). These cell lines have been authenticated by short tandem repeat (STR) profiling. GenePrint® 24 System (Promega, Madison, WI, USA) was used as the reagent. Analysis was performed according to the attached manufacturer’s protocol. Applied Biosystems 3730xl DNA analyzer (Thermo Fisher Scientific Inc., Waltham, MA, USA) was used for the electrophoresis device.

HeLa, in which HPV18 is integrated into chromosome 8 (8q24.21) with increased expression of MYC ^2,3^, was used as a positive control. HPV types and known integration sites of these cell lines were summarized in Table S4. As a MYC inhibitor, we used MYCi975 (S8906, Selleckchem, Houston, TX, USA), a small-molecule MYC inhibitor which engages MYC inside cells, disrupts MYC/MAX dimers, impairs MYC-driven gene expression, and increases proteasome-mediated MYC degradation.^4^ Cells were plated into PrimeSurface 96U (Sumitomo Bakelite, Tokyo, Japan) with the concentration of 5 × 10^3^ cells/well in triplicate. At 48 h after plating, MYCi975 was dispensed in five serially diluted doses from 0.1 to 100 μM and analyzed in triplicate following 96 h of drug incubation.

**Analysis of cell cycle by flow cytometry**

Matrigel was spread on a 12-well plate and the organoids were dissociated into single cells using Accumax applied at a concentration of 1×10^5^ cells/ml. Two days later, the Matrigel was overlaid, and the cells were treated with 10 nM trametinib or 1, 10, 100 μM of MYC inhibitor. After 72 h, the cells were fixed in cold 70% ethanol, treated with ribonuclease, and stained in the dark with 50 μg/ml propidium iodide (Sigma-Aldrich, St. Louis, MO, USA) at 4 °C for 15 min. Cell cycle distribution was analyzed using flow cytometry with an Epics XL instrument (Beckman Coulter, Brea, CA, USA) and BD Cell Quest Pro software (version 6.1; BD Bioscience, Franklin Lakes, NJ, USA), followed by data analysis using FlowJo software (version 10.4.2:Tree Star, Inc. Ashland, OR, USA).

**Establishment of organoid-derived xenograft models and drug sensitivity testing *in vivo***

Female 8-week-old immunodeficient BALB/cAJcl-nu/nu nude mice were purchased from CLEA Japan Inc. (Tokyo, Japan). Mice were housed together in individually ventilated cages with less than five mice per cage. All mice were maintained on a regular diurnal lighting cycle (12:12 light:dark) with ad libitum access to food and water. Wood shavings were used as bedding. organoids corresponding to 5×10^5^ cells were resuspended in 100 μL of advanced DMEM/F12 mixed with 100 μL of Matrigel and injected into the right side of the dorsal skin of mice.^5^ The tumor volume was calculated using the following formula: Volume = (width)^2^ × length/2 (length represents the largest tumor diameter and width represents the perpendicular tumor diameter). In 2/18 mice, the implanted organoids did not grow, while in the remaining 16 mice, treatment was initiated when the tumor volume reached approximately 100 mm^3^. The mice were alternately assigned to two groups (trametinib: n = 8; control: n = 8) in order of xenograft size at the beginning of the treatment. The median tumor volumes and weights at the beginning of the treatment and days after implantation in each group were statistically analyzed by Wilcoxon rank-sum test. Each mouse was treated 6 days/week for up to 25 days with either an oral dose of 1 mg/kg trametinib or vehicle. The tumor size and body weight of the mice were measured twice per week after treatment initiation. Data were accumulated until either the tumor volume exceeded 500 mm^3^ or the duration of treatment reached 25 days, whichever came first. The statistical significance of the Kaplan–Meier survival curves was determined using a log-rank test. Statistical significance was set at *P*<0.05. Statistical analyses were performed using JMP Pro, version 16 (SAS).

**References**

1. Picelli S, Faridani OR, Björklund AK, Winberg G, Sagasser S, Sandberg R. Full-length RNA-seq from single cells using Smart-seq2. Nat Protoc. 2014;9(1):171–181. doi: 10.1038/nprot.2014.006.
2. Shen C, Liu Y, Shi S, et al. Long-distance interaction of the integrated HPV fragment with MYC gene and 8q24.22 region upregulating the allele-specific MYC expression in HeLa cells. Int J Cancer. 2017;141(3):540–548. doi:10.1002/ijc.30763
3. Adey A, Burton JN, Kitzman JO, et al. The haplotype-resolved genome and epigenome of the aneuploid HeLa cancer cell line. Nature. 2013;500(7461):207–211. doi:10.1038/nature12064
4. Han H, Jain AD, Truica MI, et al. Small-molecule MYC inhibitors suppress tumor growth and enhance immunotherapy. Cancer Cell. 2019;36(5):483–497.e15. doi:10.1016/j.ccell.2019.10.00153..
5. Maru Y, Tanaka N, Ebisawa K, et al. Establishment and characterization of patient-derived organoids from a young patient with cervical clear cell carcinoma. Cancer Sci. 2019;110(9):2992–3005. doi:10.1111/cas.14119
