# Supplementary figures and images for: Application of organoid culture from HPV18‐positive small cell carcinoma of the uterine cervix for precision medicine
Source: Cancer Med. 2023 Jan 23;12(7):8476–89. doi: 10.1002/cam4.5588 (PMC10134306; doi:10.1002/cam4.5588)

Fig. S1

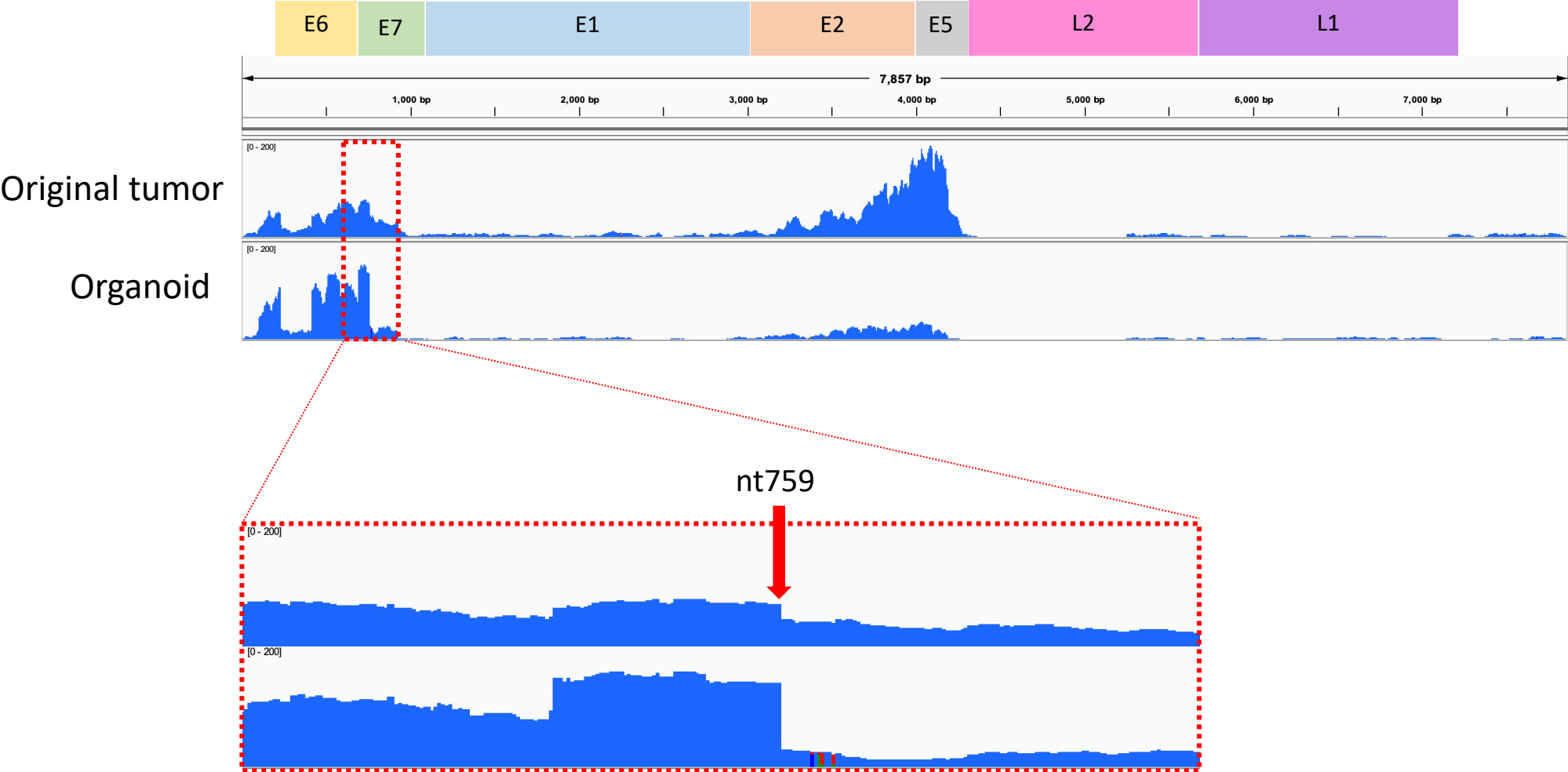

Supplement: Supplementary file 2 — Figure S1. [file CAM4-12-8476-s001.pdf]
